# Association between metabolic syndrome and risk of benign prostatic hyperplasia: a prospective cohort study of 163 975 participants

**DOI:** 10.7189/jogh.15.04275

**Published:** 2025-10-03

**Authors:** Jiaming He, Xinkai Pan, Dingwen Liu, Jiaren Li, Youyou Li, Yichuan Wang, Jinjing Guo, Qing Zhou, Liang Zhou, Long Wang

**Affiliations:** 1 Department of Urology, The Third Xiangya Hospital of Central South University, Changsha, China; 2 Department of Andrology, The First Hospital of Hunan University of Chinese Medicine, Changsha, China

## Abstract

**Background:**

Previous studies have identified metabolic syndrome (MetS) as a risk factor for benign prostatic hyperplasia (BPH). However, large-scale evidence remains to be established. This study aims to examine the association between MetS and the risk of incident BPH using large-scale cohort data, as well as the underlying biological mechanisms.

**Methods:**

We used prospective cohort data from the UK Biobank, including 163 975 male participants. We applied Cox proportional hazards models to estimate the risk of BPH associated with MetS, adjusting for sociodemographic and lifestyle factors. A mediation analysis was conducted to investigate the potential mediating role of various biomarkers.

**Results:**

The median follow-up period was 13.40 years, during which 6614 participants (12.03%) with MetS developed BPH. The presence of MetS was associated with an increased risk of BPH (hazard ratio = 1.07; 95% confidence interval = 1.03–1.10). Further analysis showed that longer follow-up duration, individual MetS components (elevated waist circumference, elevated triglycerides, elevated blood pressure, elevated glycated haemoglobin, and reduced high-density lipoprotein cholesterol), and the cumulative number of components were all significantly associated with an increased risk of BPH. Mediation analysis indicated that inflammation, erythrocyte-related biomarkers, liver function, and renal function partially mediated these associations.

**Conclusions:**

MetS is a significant risk factor for the incident BPH. Inflammation, erythrocyte-related biomarkers, liver and renal function partially mediate this relationship. Early detection and intervention in MetS may help reduce the risk of developing BPH.

Benign prostatic hyperplasia (BPH) is a prevalent urological condition that affects approximately 50% of men over 60s and 80% of those over 80s [1]. It frequently causes bladder outlet obstruction (BOO) and lower urinary tract symptoms (LUTS), significantly reducing quality of life [2]. Numerous epidemiological studies have identified components of metabolic syndrome (MetS) as potential risk factors for BPH [3–5]. Evidence from a prospective study suggests that MetS may promote the clinical progression of BPH [6]. While several studies have confirmed this association, the specific metabolic components and the potential mechanisms mediating these effects remain incompletely understood [7].

MetS refers to a cluster of metabolic abnormalities, including elevated blood pressure, central obesity, dyslipidaemia, and hyperglycaemia [8]. Driven by changes in lifestyle and dietary habits, its prevalence has risen sharply, with over one billion people worldwide currently affected [9]. The underlying overlapping pathophysiological mechanisms linking MetS and BPH are believed to involve hyperinsulinemia/insulin resistance, dysregulated adipokines, and other related factors [10]. While the association between MetS and BPH has been established in prior literature, few studies have systematically explored the temporality of this relationship or the potential mediating role of metabolic biomarkers using prospective cohort data. Moreover, the precise biological pathways linking BPH and MetS remain poorly understood. Previous research has identified correlations between BPH and several biomarkers, including those associated with the liver [11–13], kidney [14,15], erythrocytes [16], and especially, inflammation [17–21]. These biomarkers are closely associated with the development of MetS. However, there is a lack of large-scale prospective studies that explore whether, and to what extent, these biomarkers mediate the relationship between BPH and MetS.

The aim of this study is to utilise data from the UK Biobank, a large-scale prospective cohort, to analyse the association between MetS and the risk of BPH. We also performed mediation analyses to explore the extent to which systemic inflammation, liver and kidney function, and erythrocyte-related biomarkers may mediate this relationship.

## METHODS

### Study population

The study population was drawn from the UK Biobank, a large-scale prospective cohort study with more than 500 000 participants aged 37–73 years, recruited between 2006 and 2010 across the UK [22]. The UK Biobank provides comprehensive health data, including physical measurements, blood and urine biomarkers, and detailed self-reported health and lifestyle information collected at baseline. Follow-up data on health outcomes, including hospital admissions and death registries, are updated. The schematic representation of our study is shown in Figure S1 in the **Online Supplementary Document**.

The study population included male participants aged 40–69 years at baseline, with available data on relevant exposures and outcomes. To focus on incident cases of BPH, individuals with self-reported or clinically diagnosed BPH prior to baseline were excluded to minimise potential reverse causation. To examine the mediating effects of inflammatory markers in the relationship between MetS and BPH, participants without biomarker data or with extreme outlier values were excluded. This resulted in a subpopulation of individuals with complete biomarker data (Figure S2 in the **Online Supplementary Document**).

### Assessment of metabolic syndrome

The definition of MetS was based on the 2005 criteria proposed by the National Cholesterol Education Program Adult Treatment Panel III (NCEP ATP III) [23] and previous studies [24,25]. A diagnosis of MetS requires the presence of at least three of the following five components:

1) central obesity: waist circumference (WC)>88 cm for women and >102 cm for men;

2) elevated triglycerides (TG): serum TG>1.7 mmol/L;

3) reduced high-density lipoprotein (HDL) cholesterol: HDL<1.03 millimoles per litre (mmol/L) in men or <1.29 mmol/L in women, or use of lipid-lowering medications. Since lipid-lowering medications can concurrently affect multiple lipid fractions – including both HDL cholesterol and triglycerides – we categorised them under the reduced HDL cholesterol component to minimise potential double counting [26,27];

4) elevated blood pressure: a previous diagnosis of hypertension, systolic BP≥130 millimetres of mercury (mmHg) or diastolic BP≥85 mm Hg, or use of antihypertensive medications;

5) hyperglycaemia: a diagnosis of type 2 diabetes mellitus, the use of insulin, or glycated haemoglobin (HbA1c)≥42 mmol/mol, used as a surrogate for fasting glucose due to its unavailability in UK Biobank, in line with American Diabetes Association (ADA) recommendations [28].

MetS was assessed based on baseline data collected during the initial UK Biobank assessment visit. To ensure temporal precedence and avoid time-dependent misclassification, MetS status was not updated during follow-up, in line with standard practice in prospective cohort studies.

### Assessment of benign prostatic hyperplasia

BPH cases were identified using the International Classification of Diseases, 10th Revision (ICD-10) code N40 recorded in the ‘First Occurrence’ fields provided by the UK Biobank, representing the earliest date a diagnosis was captured across multiple health-related data sources, including hospital admissions, primary care records, and death registries. Additionally, we utilised self-reported data from participants who explicitly reported ‘hyperplasia of prostate’ through the UK Biobank verbal interview (Field 135). Combining ICD-10 coding and self-report maximised diagnostic specificity and reduced misclassification from LUTS due to other urological conditions.

### Assessment of mediators

To identify potential mediators in the relationship between MetS and BPH, we selected blood biomarkers that have been previously associated with inflammation, erythrocyte count, and liver and renal function. The biomarkers were selected based on existing evidence of relevant pathways. Inflammation included the leukocyte count, neutrophil count and percentage, monocyte count and percentage, lymphocyte count and percentage, platelet count, mean platelet volume and C-reactive protein (CRP). Erythrocyte function markers included red blood cell (RBC) count, reticulocyte count, RBC distribution width, haematocrit percentage, and haemoglobin concentration. Liver function markers included alanine aminotransferase (ALT), alkaline phosphatase (ALP), aspartate aminotransferase (AST), gamma-glutamyltransferase (GGT), total bilirubin (TBIL), total protein (TP) and albumin. Renal function markers included cystatin C, urate, urea and creatinine. To ensure data quality and minimise the influence of biologically implausible values, we excluded extreme outliers for each biomarker based on the distribution. Outliers were defined as those falling outside the range of the first and third quartiles plus or minus three interquartile ranges (Q1 – 3 × IQR or Q3 + 3 × IQR).

In addition to the individual inflammatory biomarkers mentioned earlier, several composite inflammatory indices that have been widely used in previous clinical and epidemiological studies were also included, such as the low-grade chronic inflammation score (INFLA) [29,30], systemic immunoinflammatory index (SII) [31,32], neutrophil-to-lymphocyte ratio (NLR) [33], platelet-to-lymphocyte ratio (PLR) and monocyte-to-lymphocyte ratio (MLR) [34]. These indices provide a comprehensive assessment of the impact of inflammation on the relationship between MetS and BPH. The INFLA score and SII are calculated using lymphocyte, neutrophils, platelets, NLR, and CRP. The INFLA score is calculated by assigning values to each biomarker based on its percentile rank. Specifically, biomarkers within the highest deciles (7th to 10th) are assigned scores ranging from +1 to +4, reflecting elevated levels of inflammation. Conversely, biomarkers in the lowest deciles (1st to 4th) are assigned values between −4 and −1, indicating lower levels of inflammation. The INFLA score ranges from −16 to +16, with higher scores corresponding to greater degrees of low-grade chronic inflammation [35]. The SII was calculated as platelet count × neutrophil count / lymphocyte count.

### Assessment of covariates

Several covariates were included in the analysis to control for potential confounders that may influence the association between MetS and BPH. The sociodemographic factors collected included age, ethnicity (‘white’, ‘non-white’), body mass index (BMI; ‘underweight’ –<18.5 kg/m^2^, ‘normal’ – 18.5 to <25, ‘overweight’ – 25 to <30, ‘obese’ –≥30), the Townsend deprivation index [36], and socioeconomic status (SES). Baseline age was calculated using the date of birth and the date of attendance at the assessment centre. The Townsend deprivation index was derived from the participants’ residential postal codes and categorised into quartiles, ranging from 1 (least deprived) to 4 (most deprived). SES was derived through a calculation that incorporated three key factors: average total household income, qualifications, and current employment status. It was then categorised into three levels: ‘low SES’, ‘mid SES’, and ‘high SES’ [37].

Lifestyle factors were also collected, including smoking status (‘smoking’, ‘non-smoking’), drinking status (‘drinking’, ‘non-drinking’), physical activity, and healthy diet score. Physical activity was assessed using metabolic equivalent task (MET) minutes per week, calculated based on adapted International Physical Activity Questionnaire (IPAQ) guidelines as implemented by the UK Biobank [38]. Total MET values reflect combined time spent in walking, moderate, and vigorous activity. Participants were classified as ‘non-enough’ (≤600 MET-min/week) or ‘enough’ (>600 MET-min/week) by standard thresholds. The healthy diet score was based on the intake of various food groups, including fruits, vegetables, fish, dairy products, oil, fine grains, sugary drinks, and processed and unprocessed meat. Fruits, vegetables, fish, dairy products, and oil were assigned scores ranging from 0 (‘non-enough’) to 1 (‘enough’), while fine grains, sugary drinks, and processed and unprocessed meat were scored from 0 (‘high intake’) to 1 (‘low intake’) [37,39]. The overall diet score was calculated by summing the individual scores and then categorised into two groups (‘healthy diet’ – score ≥5, ‘non-healthy diet’ –<5) (Table S1 in the **Online Supplementary Document**).

### Statistical analysis

Prior to analysis, we examined the distribution of continuous variables using Q–Q plots, which indicated approximate symmetry with minor deviations from normality. Accordingly, we reported continuous variables as mean ± standard deviation (SD) for descriptive purposes, while using Wilcoxon rank-sum tests for group comparisons. Categorical variables were summarised as frequencies and percentages and compared using Pearson χ^2^ tests. Kaplan-Meier curves were constructed to estimate the cumulative incidence of BPH in participants with and without MetS. Log-rank tests were employed to assess the existence of any significant differences between the survival curves. Cox proportional hazards regression models were used to examine the relationships between BPH and MetS, controlling for potential confounding variables in three models. In Model 1, no covariates were adjusted for. In Model 2, the variables were adjusted for age, ethnicity, SES, and the Townsend deprivation index. In Model 3, additional adjustments were made for BMI, smoking status, drinking status, physical activity, and healthy diet score.

The objective of the mediation analysis was to determine whether specific biomarkers mediate the relationship between MetS and BPH. The analysis used the bootstrap method with 1000 resamples to estimate percent mediated (PM) and calculate the 95% confidence intervals (CIs) (‘mediation’ package in R software). Initially, multiple linear regression models were utilised to assess the correlation between MetS and each biomarker. Then, Cox regression models were utilised to assess the relationship between BPH and these biomarkers. Only those biomarkers showing significant associations in both analyses were included in the mediation analysis. To ensure comparability across different biomarkers, raw data were standardised to z-scores. The mediation model was adjusted for the same covariates as in Model 3.

To assess the robustness of the findings, the following sensitivity analyses were conducted:

1) participants diagnosed with BPH within the first three years of follow-up were excluded to reduce reverse causation risk;

2) MetS was redefined using the International Diabetes Federation (IDF) criteria to ascertain whether the observed associations were consistent across different definitions [40,41];

3) Cox proportional hazards models were rerun using age as the underlying time scale instead of time.

All analyses were conducted using R software, version 4.2.3 (*R* Foundation for Statistical Computing, Vienna, Austria). In the prospective association analysis, a two-tailed *P*-value <0.05 was considered significant. In the context of biomarker analyses, a false discovery rate (FDR)-adjusted *P*-value <0.05 was considered significant.

## RESULTS

### Baseline characteristics

**Table 1** presents the baseline characteristics of participants stratified by the presence of BPH. Among the 163 975 participants, 17 675 (10.8%) developed BPH during follow-up. Compared with participants without BPH, those with BPH were older at enrolment, and were more likely to have lower SES, be smokers, and report poorer dietary habits. Additionally, participants with BPH had higher BMI and were more likely to exhibit elevated waist circumference, blood pressure, and HbA1c, as well as reduced HDL-C levels. The use of cholesterol-lowering and blood pressure medications was also more common among those with BPH. Participant characteristics stratified by MetS status are presented in Table S2 in the **Online Supplementary Document**.

**Table 1:** Baseline characteristics by benign prostatic hyperplasia status (n = 163 975)

Characteristic	No BPH (n = 146 300)	BPH (n = 17 675)	Overall (n = 163 975)	*P-*value*
Age at enrolment, x̄ ± SD	55.76 ± 8.20	60.63 ± 6.30	56.29 ± 8.16	<0.001
Follow-up length, x̄ ± SD	13.10 ± 2.23	7.47 ± 3.99	12.50 ± 3.04	<0.001
Ethnicity, n (%)				<0.001
*White*	137 749 (94.16)	16 796 (95.03)	154 545 (94.25)	
*Non-white*	8551 (5.84)	879 (4.97)	9430 (5.75)	
Townsend deprivation index, n (%)				<0.001
*1 (least deprived)*	35 448 (24.23)	4493 (25.42)	39 941 (24.36)	
*2*	35 648 (24.37)	4596 (26.00)	40 244 (24.54)	
*3*	36 224 (24.76)	4242 (24.00)	40 466 (24.68)	
*4 (most deprived)*	38 980 (26.64)	4344 (24.58)	43 324 (26.42)	
SES, n (%)				<0.001
Low SES	32 480 (22.20)	4995 (28.26)	37 475 (22.85)	
Mid SES	83 630 (57.16)	9207 (52.09)	92 837 (56.62)	
High SES	15 084 (10.31)	1298 (7.34)	16 382 (9.99)	
Drinking status, n (%)				<0.001
*Drinking*	76 168 (52.06)	8514 (48.17)	84 682 (51.64)	
*Non-drinking*	70 132 (47.94)	9161 (51.83)	79 293 (48.36)	
Smoking status, n (%)				<0.001
*Smoking*	75 356 (51.51)	9858 (55.77)	85 214 (51.97)	
*Non-smoking*	70 151 (47.95)	7709 (43.62)	77 860 (47.48)	
Physical activity level, n (%)				0.070
*Enough physical activity*	101 006 (69.04)	12085 (68.37)	113 091 (68.97)	
*Non-enough physical activity*	45 294 (30.96)	5590 (31.63)	50 884 (31.03)	
Healthy diet score, n (%)				<0.001
*Yes*	30 256 (20.68)	3895 (22.04)	34 151 (20.83)	
*No*	116 044 (79.32)	13 780 (77.96)	129 824 (79.17)	
BMI ± SD	27.81 ± 4.24	28.03 ± 4.18	27.84 ± 4.24	<0.001
Elevated WC, n (%)	39 678 (27.12)	5504 (31.14)	45 182 (27.55)	<0.001
Elevated TG, n (%)	72 976 (49.88)	8856 (50.10)	81 832 (49.91)	0.6
Elevated BP, n (%)	112 214 (76.70)	14 483 (81.94)	126 697 (77.27)	<0.001
Elevated HbA1c, n (%)	14 652 (10.02)	2314 (13.09)	16 966 (10.35)	<0.001
Reduced HDL, n (%)	52 828 (36.11)	7846 (44.39)	60 674 (37.00)	<0.001
Cholesterol lowering medication, n (%)	32 311 (22.09)	5525 (31.26)	37 836 (23.07)	<0.001
Blood pressure medication, n (%)	13 434 (9.18)	2256 (12.76)	15 690 (9.57)	<0.001
Insulin use, n (%)	2202 (1.50)	314 (1.78)	2516 (1.53)	0.006

BPH – benign prostatic hyperplasia, BMI – body mass index, HDL – high-density lipoprotein, SD – standard deviation, SES – socioeconomic status, TG – triglyceride, WC – waist circumference, x̄ – mean

*P-values were calculated using the Wilcoxon rank-sum test for continuous variables and Pearson χ^2^ test for categorical variables.

### Cumulative incidence of BPH by MetS status

The cumulative risk of BPH among participants with and without MetS is shown in Figure S3 in the **Online Supplementary Document**. During a median follow-up period of 13.40 years, 6614 participants (12.03%) with MetS and 10 288 participants (9.44%) without MetS developed BPH. The cumulative incidence of BPH at 3, 9, and 15 years was 2.38, 7.71, and 15.04%, respectively, in the MetS group and 1.79, 5.92, and 11.50% in the non-MetS group. The cumulative incidence of BPH was significantly higher in participants with MetS compared to those without MetS (*P*_log-rank_<0.0001).

### BPH risk associated with MetS across different follow-up periods

**Figure 1:**
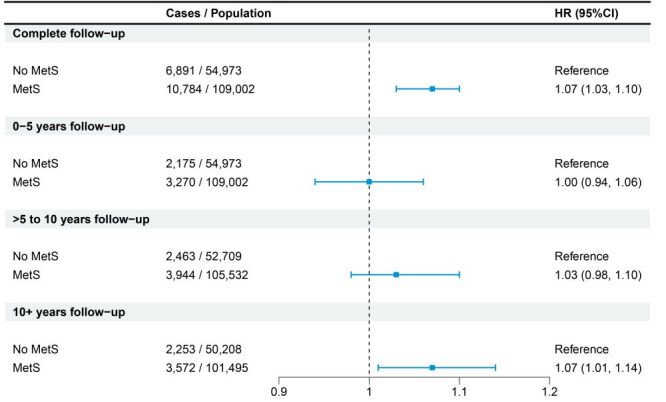
Cox proportional hazards models investigating the association between metabolic syndrome and benign prostatic hyperplasia by different follow-up periods. CI – confidence interval, HR – hazard ratio, MetS – metabolic syndrome.

The association between MetS and BPH became more pronounced over time, after adjusting for covariates in Model 3 (**Figure 1**). No significant difference in BPH risk was observed during the first five years of follow-up, while a modest increase was noted between five and 10 years. The association reached statistical significance after 10 years of follow-up, indicating a delayed effect of MetS on BPH risk.

### Effect of individual and cumulative MetS components on BPH risk

**Figure 2:**
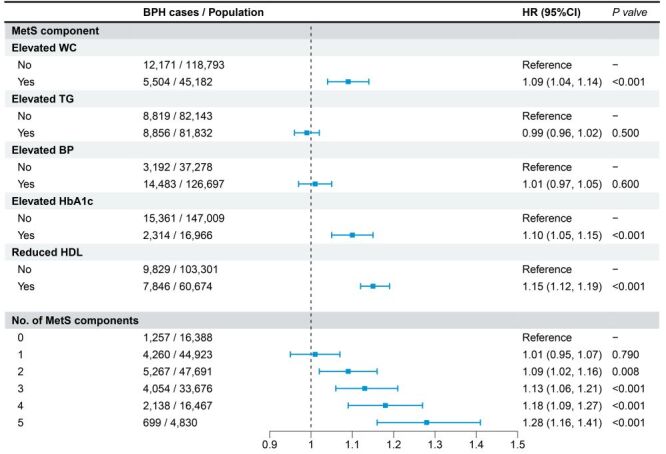
Association between individual and cumulative metabolic syndrome components and benign prostatic hyperplasia. *P*-values were derived from Cox proportional hazards models. BP – blood pressure, CI – confidence interval, HDL – high-density lipoprotein, HR – hazard ratio, MetS – metabolic syndrome, TG – triglyceride, WC – waist circumference.

As shown in **Figure 2**, the risk of BPH increased progressively with the number of MetS components, indicating a cumulative effect in the fully adjusted models (Model 3). Compared to individuals without any component, those with five had a 28% higher risk. Among the individual components, elevated waist circumference, reduced HDL-C, and elevated HbA1c were significantly associated with increased BPH risk. In contrast, elevated triglycerides and blood pressure were not significantly associated with BPH.

### Mediation analysis

The multivariate linear regression analysis (Table S3 in the **Online Supplementary Document**) showed that NLR and MLR were not significantly associated with MetS. Although several biomarkers – including monocyte count and percentage, reticulocyte count, ALT, ALP, CRP, platelet and leukocyte count, mean platelet volume, and PLR – were significantly correlated with MetS, they were not associated with BPH in the Cox model. Haematocrit and haemoglobin, despite their initial associations with both MetS and BPH, were no longer significant after FDR adjustment.

**Figure 3:**
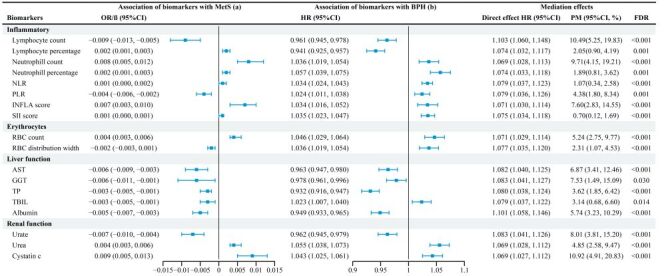
Association of benign prostatic hyperplasia with metabolic syndrome mediated by biomarkers. Associations between metabolic syndrome and biomarkers (a-path) estimated using multivariable linear regression models, adjusted for covariates in Model 3. Cox proportional hazards models evaluating associations between biomarkers and benign prostatic hyperplasia (b-path), and estimating direct and indirect (mediation) effects. *P*-values are adjusted for false discovery rate (FDR). Mediation significance was evaluated using bootstrap methods with 1000 resamples. AST – aspartate aminotransferase, BPH – benign prostatic hyperplasia, CI – confidence interval, FDR – false discovery rate, GGT – gamma-glutamyltransferase, HR – hazard ratio, MetS – metabolic syndrome, NLR – neutrophil-to-lymphocyte ratio, OR – odds ratio, PLR – platelet-to-lymphocyte ratio, PM – percent mediated, RBC – red blood cell, TBIL – total bilirubin, TP – total protein.

Significant mediating effects were observed across four biomarker domains (**Figure 3**; Table S4 in the **Online Supplementary Document**). Inflammatory markers, such as lymphocyte and neutrophil indices, along with composite indicators like the INFLA and SII scores, demonstrated modest to moderate mediation. Erythrocyte-related biomarkers, including RBC count and distribution width, also showed significant indirect effects. Liver function markers – including AST, GGT, and albumin – partially mediated the association, while renal function biomarkers, particularly cystatin C, exhibited the strongest mediating effect (10.92%).

### Sensitivity analysis

The results of the sensitivity analyses remained consistent after the following modifications were made:

1) excluding participants diagnosed with BPH within the first three years of follow-up;

2) redefining MetS using the IDF criteria;

3) using age as the underlying time scale in the Cox models (Table S5–7 in the **Online Supplementary Document**).

## DISCUSSION

In this large-scale prospective cohort study of 163 975 men, we found that individuals with MetS had a significantly higher cumulative incidence of BPH compared to those without MetS. This association became particularly pronounced after 10 years of follow-up. Furthermore, both individual MetS components and the cumulative number of components were independently associated with an increased risk of BPH. Mediation analyses revealed potential biological pathways that partially explained the relationship between MetS and BPH.

The association between BPH and MetS has been suggested by some studies. A recent meta-analysis reported that patients with MetS exhibited a higher annual prostate growth rate [42]. This finding was further supported by a cross-sectional study by Suarez Arbelaez et al., which analysed a comprehensive multi-institutional database from the United States [4]. In their study, dyslipidaemia showed a strong correlation with prostate growth, consistent with our findings. Similarly, a study based on the China Health and Retirement Longitudinal Study (CHARLS) reported that among 4588 elderly Chinese men, the risk of BPH was 1.60 times higher in individuals with MetS compared to those without MetS [43]. However, most of these studies relied on small sample sizes or used cross-sectional or retrospective designs, which limit their generalisability. In contrast, our study is the first to employ a large-scale prospective cohort to explore this association.

The correlation between the number of MetS components and the risk of BPH reinforces the evidence that individuals with a greater number of MetS components face a higher risk of developing BPH. However, it is noteworthy that the specific MetS components associated with increased BPH risk have varied across studies. For instance, Suarez Arbelaez et al. [4] reported that each MetS component was significantly associated with BPH, whereas Yang Xiong et al. [43] found no significant correlation between hypertension and BPH. In our study, no statistically significant relationship was observed between elevated triglycerides, elevated blood pressure, and BPH risk, a finding that contrasts with some prior studies. Nevertheless, the roles of elevated waist circumference and dyslipidaemia in BPH development are well supported by existing evidence, highlighting their potential central role in BPH pathogenesis.

Although MetS has been established as a risk factor for BPH in several studies, limited research has explored the underlying molecular mechanisms. Our mediation analysis identified inflammation, erythrocyte-related biomarkers, and liver and renal function-related biomarkers as potential mediators of the relationship between MetS and BPH. The significant mediating effects of inflammatory markers – such as lymphocyte count and percentage, neutrophil count and percentage, and the INFLA and SII scores – highlight the role of systemic chronic inflammation as a critical factor in the pathway linking MetS to BPH. Previous studies have documented elevated levels of pro-inflammatory cytokines in BPH tissue [44–46]. In patients with MetS, excessive fat intake may stimulate interleukin 6 (IL-6) expression [47] and induce tumour necrosis factor alpha (TNF-α) up-regulation in the prostate, thereby promoting prostate growth. Additionally, erythrocyte-related metabolites, including RBC count and RBC distribution width, appear to mediate the MetS-BPH association. Prior research has shown that elevated RBC distribution width is significantly associated with an increased risk of BPH, potentially due to high IL-6 levels in patients with MetS [16]. Elevated IL-6 levels have been linked to increased RBC distribution width, which may indirectly influence BPH development. Furthermore, our study revealed that renal and liver function biomarkers partly mediate the association between MetS and BPH [11,48]. Interestingly, elevated uric acid levels were associated with a reduced risk of BPH [14,15], consistent with previous findings. While the role of renal function in BPH pathogenesis remains underexplored, further research is needed to clarify specific mechanisms underlying the association between BPH and renal function. The relationship between liver function and BPH, however, has been more extensively studied. It is hypothesised that liver dysfunction may influence BPH development by modulating inflammatory responses and metabolic pathways. Nevertheless, further investigation is necessary to elucidate the precise mechanisms through which liver function, within the context of MetS, impacts BPH. Such insights may uncover potential clinical applications for managing BPH in patients with MetS.

There are several strengths in this study. First, we took advantage of the large cohort size and prospective design of the UK Biobank. Second, although causality cannot be definitively established, the prospective design of this cohort study, along with the careful adjustment for confounding factors, enhances the plausibility of a causal relationship between MetS and BPH. Third, the comprehensive nature of the UK Biobank data allowed us to carefully adjust for a wide range of potential confounders. Finally, we conducted a series of sensitivity analyses to ensure the robustness and reliability of our findings.

Several limitations exist in our study. First, while the UK Biobank provides a wealth of data, it is primarily based on a UK population, which may limit the generalisability of the findings to other ethnic or geographic groups. Second, the observational nature of the study precludes any definitive conclusions about causality. Although we adjusted for a range of potential confounders, residual confounding remains a possibility. Third, data on some aspects of prostate health, such as the severity of BPH or treatments received, were not available, which could affect the interpretation of the findings. Fourth, both MetS components and circulating biomarkers were assessed only once at baseline. The lack of repeated measurements prevents evaluation of intra-individual variability, potentially weakening mediation pathway robustness and introducing misclassification. Moreover, MetS status was only assessed at baseline and not updated during follow-up. Consequently, individuals who developed MetS after the baseline assessment were still classified as non-exposed, potentially introducing non-differential misclassification that could attenuate the true association. Finally, while these biomarkers provide valuable insights into MetS–BPH mechanisms, they may not capture the full complexity of the biological pathways involved.

## CONCLUSIONS

In conclusion, this large cohort study demonstrates that MetS is a significant risk factor for BPH. Further analysis showed that longer follow-up, individual MetS components, and their cumulative number were significantly associated with increased BPH risk. Our mediation analysis suggests that inflammation, erythrocyte-related biomarkers, liver and kidney function partially mediate this relationship. These findings emphasise the importance of early MetS detection and management to reduce BPH risk. Further studies are needed to explore underlying mechanisms and potential interventions.

## Additional material


Online Supplementary Document
