# Supplementary material for: Association between metabolic syndrome and risk of benign prostatic hyperplasia: a prospective cohort study of 163 975 participants
Source: J Glob Health. 2025 Oct 3;15:04275. doi: 10.7189/jogh.15.04275 (PMC12491909; doi:10.7189/jogh.15.04275)
Supplement: Online Supplementary Document [file jogh-15-04275-s001.pdf]

Supplement to: He J, Pan X, Liu D, Li J, Li Y, Wang Y, Guo J, Zhou Q, Zhou L, Wang L. Association between metabolic syndrome and risk of benign prostatic hyperplasia: a prospective cohort study of 163 975 participants. J Glob Health. 2025;15:04275.

Jiaming He[1], Xinkai Pan[1], Dingwen Liu[1], Jiaren Li[1], Youyou Li[1], Yichuan Wang[1], Jinjing Guo[1], Qing Zhou[2], Liang Zhou[1]†, Long Wang[1]†

[1]Department of Urology, The Third Xiangya Hospital of Central South University, Changsha, China

[2]Department of Andrology, The First Hospital of Hunan University of Chinese Medicine, Changsha, China

†Equal contribution.

Figure S1. Study design.

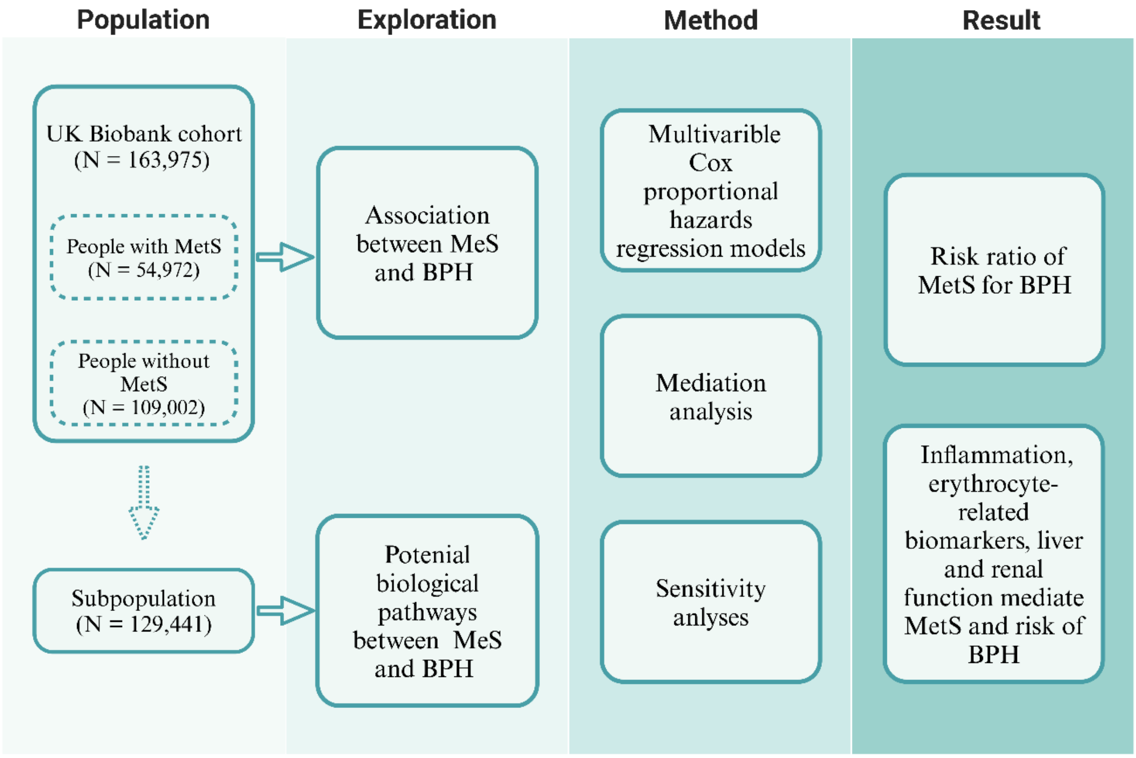

Figure S2. Cohort Flow Diagram.

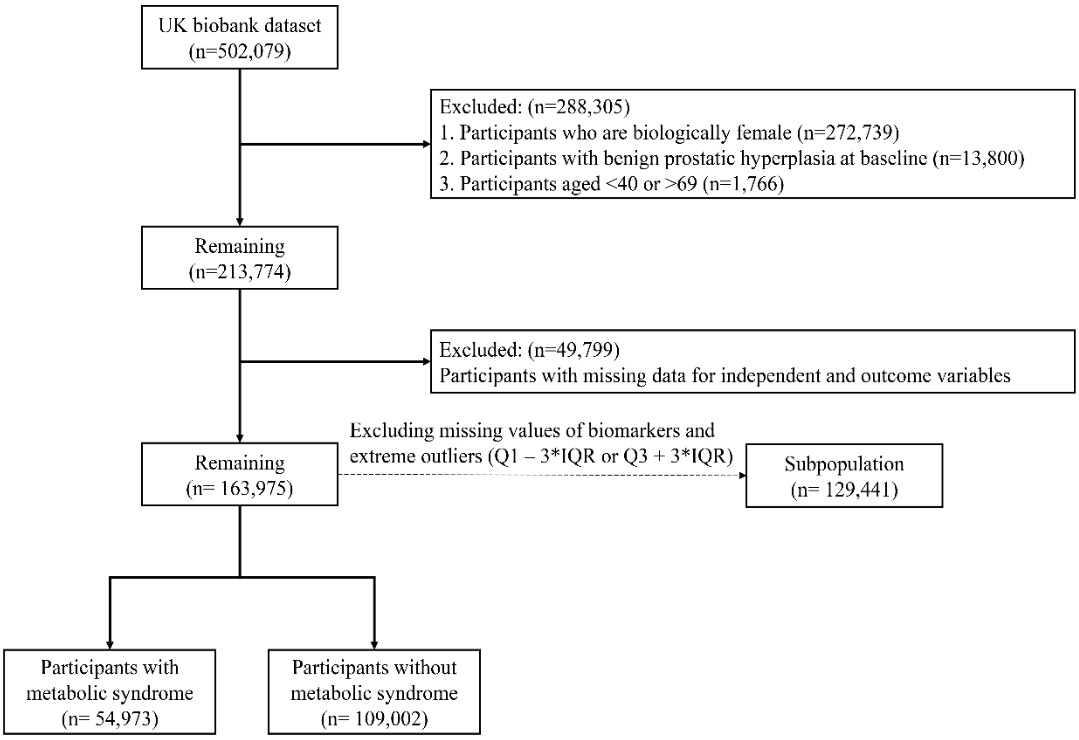

Table S1. The definition of covariates.

Covariate		Field ID	Description	Categories from raw data
	Age	21022	Age at recruitment	Continuous, years
	Ethnicity	21000	Ethnic background	White<br>Non-white
	Body mass index	21001	Body mass index	Underweight - <18.5 kg/m2<br>Normal weight - 18.5 to <25 kg/m2<br>Overweight - 25 to <30 kg/m2<br>Obese - ≥30 kg/m2
	Townsend deprivation index	22189	Townsend deprivation index at recruitment	1 (least deprived) - -6.258260 to < -3.681640<br>2 - -3.681640 to < -2.218460<br>3 - -2.218460 to < 0.368979<br>4 (most deprived) - ≥0.368979
Socioeconomic status	Income	738	Average total household income before tax	Low ses<br>Mid ses<br>High ses
	Qualifications	6138	Qualifications	Not willing to answer
	Employment	6142	Current employment status	
	Smoking status	1239	Current tobacco smoking	Smoking<br>Non-smoking
		1249	Past tobacco smoking	
		2644	Light smokers, at least 100 smokes in lifetime	
	Drinking status	1568	Average weekly red wine intake	Drinking<br>Non-drinking
		1578	Average weekly champagne plus white wine intake	
		1588	Average weekly beer plus cider	

			intake	
		1598	Average weekly spirits intake	
		1608	Average weekly fortified wine intake	
		5364	Average weekly intake of other alcoholic drinks	
	Physical activity	22038	Metabolic Equivalent Task minutes per week for moderate activity	Enough physical activity - ≥600 minutes<br>Non-Enough physical activity - <600 minutes
		22039	MET minutes per week for vigorous activity	
Healthy diet	Fruit	1309	Fresh fruit intake	Healthy diet - score ⩾ 5<br>Non-healthy diet - score < 5
		1319	Dried fruit intake	
	Vegetable	1289	Cooked vegetable intake	
		1299	Salad / raw vegetable intake	
	Whole grain	1438	Bread intake	
		1448	Bread type	
		1458	Cereal intake	
		1468	Cereal type	
	Sea food	1329	Oily fish intake	
		1339	Non-oily fish intake	
	Dairy product	1408	Cheese intake	
		1418	Milk type used	
	Oil	1428	Spread type	
		2654	Non-butter spread type details	
	Fine grain	1438	Bread intake	

	1448	Bread type
	1458	Cereal intake
	1468	Cereal type
Processed meat	1349	Processed meat intake
Unprocessed meat	1359	Poultry intake
	1369	Beef intake
	1379	Lamb/mutton intake
	1389	Pork intake
Sugary drink	6144	Never eat eggs, dairy, wheat, sugar

Table S2. Baseline characteristics by metabolic syndrome status (n=163,975).

**Characteristic**	**No MetS (N=109,002)**	**MetS (N=54,973)**	**Overall (N=163,975)**	***P* value**
Age at enrollment, Mean ± SD	55.45 ± 8.23	57.95 ± 7.75	56.29 ± 8.16	**<0.001**
Follow-up length, Mean ± SD	12.66 ± 2.87	12.18 ± 3.31	12.50 ± 3.04	**<0.001**
Ethnicity, n (%)				**<0.001**
White	103,078 (94.57)	51,467 (93.62)	154,545 (94.25)	
Non-white	5,924 (5.43)	3,506 (6.38)	9,430 (5.75)	
Townsend deprivation index, n (%)				**<0.001**
1(least deprived)	27,588 (25.31)	12,353 (22.47)	39,941 (24.36)	
2	27,262 (25.01)	12,982 (23.62)	40,244 (24.54)	
3	26,915 (24.69)	13,551 (24.65)	40,466 (24.68)	
4(most deprived)	27,237 (24.99)	16,087 (29.26)	43,324 (26.42)	
SES, n (%)				**<0.001**
Low SES	21,496 (19.72)	15,979 (29.07)	37,475 (22.85)	
Mid SES	64,351 (59.04)	28,486 (51.82)	92,837 (56.62)	
High SES	12,422 (11.40)	3,960 (7.20)	16,382 (9.99)	
Drinking status, n (%)				**<0.001**
Drinking	58,257 (53.45)	26,425 (48.07)	84,682 (51.64)	
Non-Drinking	50,745 (46.55)	28,548 (51.93)	79,293 (48.36)	
Smoking status, n (%)				**<0.001**
Smoking	52,914 (48.54)	32,300 (58.76)	85,214 (51.97)	
Non-smoking	55,606 (51.01)	22,254 (40.48)	77,860 (47.48)	
Physical activity level, n (%)				**<0.001**
Enough physical activity	78,359 (71.89)	34,732 (63.18)	113,091 (68.97)	
Non-Enough physical activity	30,643 (28.11)	20,241 (36.82)	50,884 (31.03)	
Healthy diet score, n (%)				**<0.001**
Healthy diet	24,296 (22.29)	9,855 (17.93)	34,151 (20.83)	
Non-healthy diet	84,706 (77.71)	45,118 (82.07)	129,824 (79.17)	
BMI ± SD	26.35 ± 3.26	30.78 ± 4.41	27.84 ± 4.24	**<0.001**
Elevated WC, n (%)	10,007 (9.18)	35,175 (63.99)	45,182 (27.55)	**<0.001**
Elevated TG, n (%)	36,181 (33.19)	45,651 (83.04)	81,832 (49.91)	**<0.001**

Characteristic	No MetS (N=109,002)	MetS (N=54,973)	Overall (N=163,975)	*P* value
Elevated BP, n (%)	74,036 (67.92)	52,661 (95.79)	126,697 (77.27)	**<0.001**
Elevated HbA1c, n (%)	1,892 (1.74)	15,074 (27.42)	16,966 (10.35)	**<0.001**
Reduced HDL, n (%)	18,189 (16.69)	42,485 (77.28)	60,674 (37.00)	**<0.001**
Cholesterol lowering medication, n (%)	11,544 (10.59)	26,292 (47.83)	37,836 (23.07)	**<0.001**
Blood pressure medication, n (%)	9,470 (8.69)	6,220 (11.31)	15,690 (9.57)	**<0.001**
Insulin use, n (%)	398 (0.37)	2,118 (3.85)	2,516 (1.53)	**<0.001**

MetS – metabolic syndrome, BMI – body mass index, HDL – high-density lipoprotein, SD – standard deviation, SES – socioeconomic status, TG – triglyceride, WC – waist circumference.

Figure S3. Cumulative incidence of benign prostatic hyperplasia with and without metabolic syndrome.

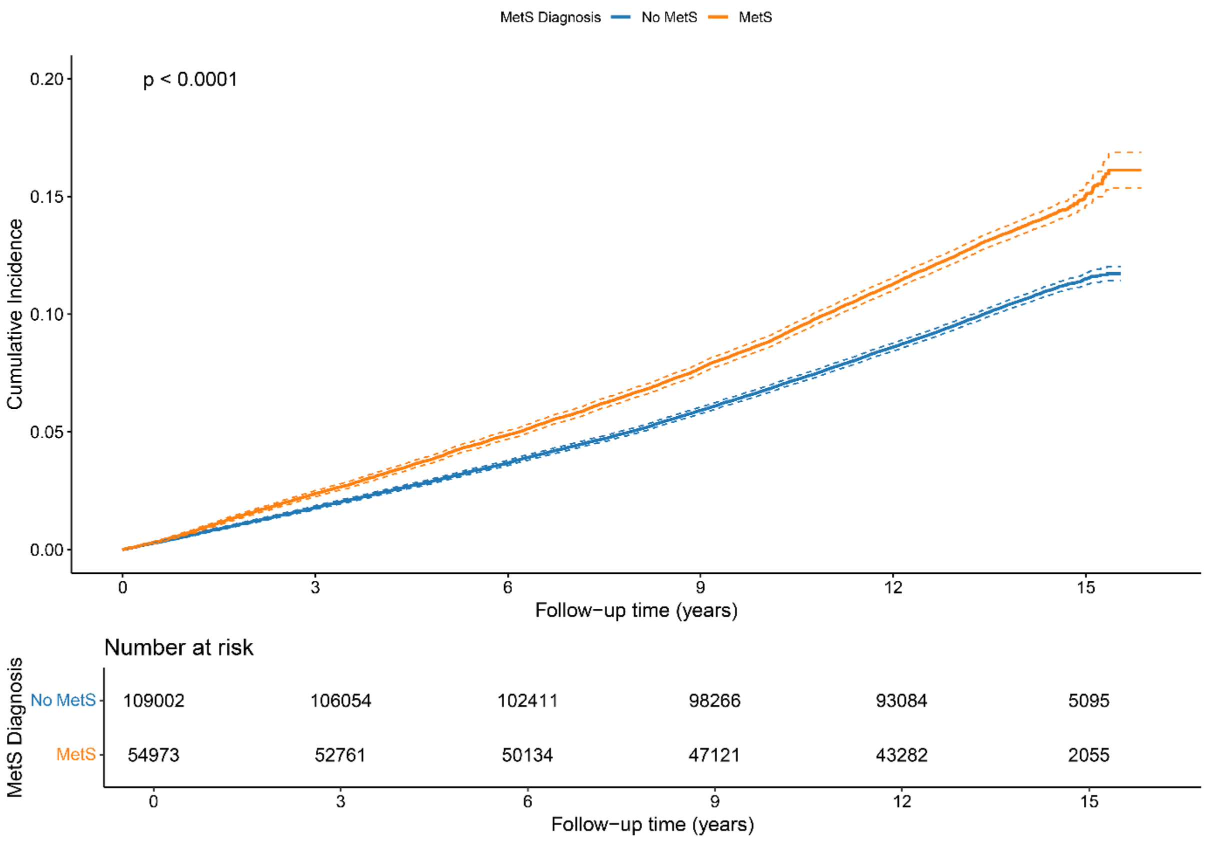

P-value was calculated using the log-rank test.

MetS – metabolic syndrome

Table S3. Mean concentration of biomarkers (n = 129,441)

Biomarkers	Unit	Mean	SD
**Inflammatory-related biomarkers**			
Leukocyte count	10^9 cells/L	6.78	1.62
Neutrophil count	10^9 cells/L	4.17	1.28
Neutrophil percentage	%	60.99	7.99
Monocyte count	10^9 cells/L	0.50	0.16
Monocyte percentage	%	7.54	2.08
Lymphocyte count	10^9 cells/L	1.88	0.56
Lymphocyte percentage	%	28.16	6.97
C reactive protein	mg/L	1.65	1.45
Platelet count	10^9 cells/L	237.28	51.21
Mean platelet volume	fL	9.29	1.06
Neutrophil-to-Lymphocyte ratio (NLR)	ratio	2.40	1.09
Platelet-to-Lymphocyte ratio (PLR)	ratio	137.14	54.00
Monocyte-to-Lymphocyte ratio (MLR)	ratio	0.28	0.12
Low-grade chronic inflammation score (INFLA) score	/	-0.03	6.00
Systemic immunoinflammatory index (SII) score	/	570.12	295.01
**Erythrocyte-related biomarkers**			
Erythrocyte count	10^12 cells/L	4.76	0.36
Reticulocyte count	10^12 cells/L	0.06	0.02
High light scatter reticulocyte count	10^12 cells/L	0.02	0.01
Red blood cell distribution width	%	13.37	0.69
Haematocrit percentage	%	43.41	2.79
Haemoglobin concentration	g/dL	15.03	0.95
**Renal function-related biomarkers**			
Urate	µmol/L	351.86	68.61
Urea	mmol/L	5.57	1.25
Cystatin C	mg/L	0.93	0.13
Creatinine	umol/L	81.00	12.10
**Liver function-related biomarkers**			
Alanine aminotransferase	U/L	25.81	10.68
Alkaline phosphatase	U/L	79.87	19.53
Aspartate aminotransferase	U/L	26.94	6.34
Gamma-glutamyl transferase	U/L	37.98	20.91
Total bilirubin	µmol/L	9.86	3.61
Total protein	g/L	72.56	3.97
Albumin	g/L	45.66	2.51

SD – standard deviation

Table S4. Selection of biomarkers between metabolic syndrome and benign prostatic hyperplasia (n = 129,441)

	Association of biomarkers with MetS(a)			Association of biomarkers with BPH(b)		
Biomarkers	*OR/β (95%CI)*	*P*	*FDR*	*HR (95%CI)*	*P*	*FDR*
**Inflammatory-related biomarkers**						
Leukocyte count, 10^9 cells/L	0.005 (-0.000, 0.010)	6.556E-02	9.691E-02	1.020 (1.002, 1.038)	2.548E-02	4.253E-02
**Neutrophil count, 10^9 cells/L**	0.008 (0.004, 0.013)	4.000E-05	1.511E-04	1.040 (1.023, 1.058)	4.657E-06	1.440E-05
**Neutrophil percentage, %**	0.002 (0.001, 0.003)	3.799E-04	9.936E-04	1.060 (1.042, 1.078)	1.871E-11	1.590E-10
Monocyte count, 10^9 cells/L	0.003 (-0.000, 0.007)	7.245E-02	1.026E-01	1.017 (1.001, 1.035)	4.326E-02	6.128E-02
Monocyte percentage, %	0.000 (-0.000, 0.000)	8.476E-01	8.733E-01	0.998 (0.981, 1.015)	8.017E-01	8.260E-01
**Lymphocyte count, 10^9 cells/L**	-0.009 (-0.013, -0.005)	7.620E-06	4.318E-05	0.961 (0.945, 0.978)	5.548E-06	1.452E-05
**Lymphocyte percentage, %**	0.002 (0.001, 0.003)	3.328E-04	9.429E-04	0.938 (0.922, 0.954)	2.679E-13	4.556E-12
C reactive protein, mg/L	0.000 (-0.002, 0.003)	7.732E-01	8.215E-01	1.006 (0.989, 1.024)	4.726E-01	5.183E-01
Platelet count, 10^9 cells/L	-0.000 (-0.000, 0.000)	9.998E-01	9.998E-01	1.001 (0.985, 1.018)	9.000E-01	9.000E-01
Mean platelet volume, fL	-0.000 (-0.001, 0.000)	4.277E-01	4.847E-01	0.994 (0.978, 1.010)	4.648E-01	5.183E-01
**Neutrophil-to-Lymphocyte Ratio (NLR)**	0.001 (0.000, 0.002)	1.098E-03	2.489E-03	1.034 (1.024, 1.043)	6.657E-13	7.548E-12
**Platelet-to-Lymphocyte Ratio (PLR)**	-0.004 (-0.006, -0.002)	5.199E-04	1.263E-03	1.025 (1.011, 1.039)	2.900E-04	5.801E-04
Monocyte-to-Lymphocyte ratio (MLR)	0.000 (-0.000, 0.001)	1.793E-01	2.345E-01	1.025 (1.015, 1.035)	7.564E-07	2.570E-06
**Low-grade chronic inflammation score (INFLA) score**	0.006 (0.003, 0.010)	1.814E-04	5.607E-04	1.039 (1.021, 1.057)	1.629E-05	3.695E-05
**Systemic immunoinflammatory index (SII) score**	0.001 (0.000, 0.001)	3.096E-02	5.263E-02	1.036 (1.024, 1.048)	9.574E-10	4.648E-09
**Erythrocyte-related biomarkers**						
**Erythrocyte count, 10^12 cells/L**	0.004 (0.003, 0.006)	6.700E-07	5.695E-06	1.046 (1.028, 1.064)	2.077E-07	8.840E-07
Reticulocyte count, 10^12 cells/L	0.003 (-0.004, 0.010)	4.169E-01	4.847E-01	1.009 (0.991, 1.027)	3.346E-01	3.922E-01
High light scatter reticulocyte count, 10^12 cells/L	0.004 (-0.004, 0.011)	2.800E-01	3.526E-01	1.011 (0.993, 1.030)	2.252E-01	2.735E-01
**Red blood cell (erythrocyte) distribution width, %**	-0.002 (-0.003, -0.001)	1.693E-04	5.607E-04	1.037 (1.020, 1.054)	1.353E-05	3.279E-05
Hematocrit percentage, %	0.001 (0.000, 0.001)	2.617E-02	4.853E-02	1.021 (1.005, 1.038)	1.180E-02	2.229E-02

	Association of biomarkers with MetS(a)			Association of biomarkers with BPH(b)		
Biomarkers	*OR/β (95%CI)*	*P*	*FDR*	*HR (95%CI)*	*P*	*FDR*
Hemoglobin concentration, g/Dl	0.001 (0.000, 0.002)	4.022E-02	6.512E-02	1.017 (1.001, 1.034)	4.316E-02	6.128E-02
**Renal function-related biomarkers**						
**Urate, μmol/L**	-0.007 (-0.010, -0.004)	1.650E-05	8.014E-05	0.960 (0.943, 0.977)	5.149E-06	1.452E-05
**Urea, mmol/L**	0.004 (0.003, 0.006)	3.110E-08	5.695E-07	1.054 (1.037, 1.072)	2.664E-10	1.809E-09
**Cystatin C, mg/L**	0.009 (0.005, 0.013)	2.140E-06	1.455E-05	1.046 (1.028, 1.064)	2.544E-07	9.596E-07
Creatinine, μmol/L	-0.000 (-0.000, 0.000)	5.289E-01	5.801E-01	1.005 (0.988, 1.021)	5.765E-01	6.126E-01
**Liver function-related biomarkers**						
Alanine aminotransferase, U/L	-0.005 (-0.012, 0.001)	1.411E-01	1.919E-01	0.985 (0.967, 1.004)	1.122E-01	1.468E-01
Alkaline phosphatase, U/L	0.001 (-0.001, 0.003)	4.235E-01	4.847E-01	1.011 (0.995, 1.028)	1.909E-01	2.403E-01
**Aspartate aminotransferase, U/L**	-0.006 (-0.009, -0.003)	2.820E-05	1.199E-04	0.963 (0.947, 0.980)	2.128E-05	4.526E-05
**Gamma-glutamyl transferase, U/L**	-0.006 (-0.011, -0.001)	1.569E-02	3.138E-02	0.980 (0.962, 0.998)	2.627E-02	4.253E-02
**Total bilirubin, μmol/L**	-0.003 (-0.005, -0.001)	7.367E-03	4.341E-06	1.021 (1.004, 1.038)	1.401E-02	2.506E-02
**Total protein, g/L**	-0.003 (-0.004, -0.002)	3.830E-07	1.565E-02	0.934 (0.918, 0.950)	2.638E-15	8.976E-14
**Albumin, g/L**	-0.005 (-0.007, -0.003)	3.350E-08	5.695E-07	0.947 (0.931, 0.964)	6.642E-10	3.763E-09

Biomarkers concentrations were standardized by z-score. (a) associations between metabolic syndrome and biomarkers (a-path) estimated using multivariable linear regression models, adjusted for covariates in Model 3. (b) Cox proportional hazards models evaluating associations between biomarkers and benign prostatic hyperplasia (b-path), and estimating direct and indirect (mediation) effects.
MetS – metabolic syndrome, BPH – benign prostatic hyperplasia, HR – hazard ratio, CI – confidence interval, OR – odds ratio, FDR – false discovery rate.

Table S5. Sensitivity analyses of excluding participants diagnosed with benign prostatic hyperplasia within the first three years of follow-up (n=160,735)

Variables	Risk of BPH					
	Model 1		Model 2		Model 3	
	**HR (95% CI)**	***P* value**	**HR (95% CI)**	***P* value**	**HR (95% CI)**	***P* value**
**MetS status**						
No MetS	1(Reference)		1(Reference)		1(Reference)	
MetS	1.32 (1.28-1.37)	<0.001	1.13 (1.08-1.16)	<0.001	1.09 (1.04-1.13)	<0.001
**No. of MetS traits**						
0	1(Reference)		1(Reference)		1(Reference)	
1	1.27 (1.18, 1.36)	<0.001	1.01 (0.94-1.08)	0.777	1.02 (0.95-1.09)	0.669
2	1.49 (1.39, 1.59)	<0.001	1.09 (1.02, 1.17)	0.001	1.09 (1.02, 1.17)	0.002
3	1.64 (1.53, 1.76)	<0.001	1.14 (1.06, 1.22)	<0.001	1.12 (1.04, 1.21)	<0.001
4	1.84 (1.70, 1.99)	<0.001	1.21 (1.12, 1.31)	<0.001	1.19 (1.09, 1.29)	<0.001
5	2.19 (1.97, 2.42)	<0.001	1.36 (1.22, 1.50)	<0.001	1.31 (1.17, 1.46)	<0.001
**Individual MetS components**						
Elevated WC	1.23 (1.19, 1.28)	<0.001	1.12 (1.08, 1.16)	<0.001	1.08 (1.03, 1.14)	0.001
Elevated TG	1.02 (0.98, 1.05)	0.316	1.03 (0.99, 1.06)	0.101	1.00 (0.97, 1.04)	0.885
Elevated BP	1.37 (1.31, 1.43)	<0.001	1.01 (0.97, 1.06)	0.605	1.00 (0.96, 1.05)	0.903
Elevated HbA1c	1.43 (1.36, 1.50)	<0.001	1.16 (1.10, 1.22)	<0.001	1.11 (1.06, 1.17)	<0.001
Reduced HDL	1.42 (1.37, 1.57)	<0.001	1.18 (1.14, 1.22)	<0.001	1.15 (1.11, 1.19)	<0.001

Model 1 adjusted for no covariates. Model 2 adjusted for age, ethnicity, smoking status, socioeconomic status, and Townsend's deprivation index. Model 3 adjusted for model 2 plus BMI, smoking status, drinking status, physical activity, and healthy diet score
HR – hazard ratio, CI – confidence interval.

Table S6. Sensitivity analyses of MetS were redefined using the International Diabetes Federatio criteria (n=163,975)

Variables	Risk of BPH					
	Model 1		Model 2		Model 3	
	HR (95% CI)	*P* value	HR (95% CI)	*P* value	HR (95% CI)	*P* value
**MetS status**						
No MetS	1(Reference)		1(Reference)		1(Reference)	
MetS	1.33 (1.29-1.37)	<0.001	1.12 (1.09-1.16)	<0.001	1.09 (1.05-1.13)	<0.001
**No. of MetS traits**						
0	1(Reference)		1(Reference)		1(Reference)	
1	1.26 (1.19, 1.35)	<0.001	1.00 (0.93-1.07)	0.968	1.01 (0.95-1.07)	0.794
2	1.50 (1.41, 1.59)	<0.001	1.09 (1.02, 1.16)	0.009	1.09 (1.02, 1.16)	0.007
3	1.67 (1.56, 1.77)	<0.001	1.14 (1.07, 1.21)	<0.001	1.13 (1.06, 1.21)	<0.001
4	1.84 (1.71, 1.97)	<0.001	1.20 (1.11, 1.28)	<0.001	1.18 (1.09, 1.27)	<0.001
5	2.15 (1.96, 2.36)	<0.001	1.31 (1.20, 1.44)	<0.001	1.28 (1.16, 1.41)	<0.001
**Individual MetS components**						
Elevated WC	1.23 (1.19, 1.27)	<0.001	1.11 (1.08, 1.15)	<0.001	1.09 (1.04, 1.14)	0.001
Elevated TG	1.00 (0.97, 1.03)	0.795	1.01 (0.99, 1.05)	0.328	0.99 (0.96, 1.02)	0.546
Elevated BP	1.39 (1.34, 1.44)	<0.001	1.02 (0.98, 1.06)	0.435	1.01 (0.97, 1.05)	0.580
Elevated HbA1c	1.42 (1.36, 1.48)	<0.001	1.15 (1.10, 1.20)	<0.001	1.10 (1.05, 1.15)	<0.001
Reduced HDL	1.43 (1.39, 1.47)	<0.001	1.19 (1.15, 1.22)	<0.001	1.15 (1.12, 1.19)	<0.001

Model 1 adjusted for no covariates. Model 2 adjusted for age, ethnicity, smoking status, socioeconomic status, and Townsend's deprivation index. Model 3 adjusted for model 2 plus BMI, smoking status, drinking status, physical activity, and healthy diet score
HR – hazard ratio, CI – confidence interval.

Table S7. Sensitivity analyses of using age as the underlying time scale instead of time (n=163,975)

Variables	Risk of BPH					
	Model 1		Model 2		Model 3	
	**HR (95% CI)**	***P* value**	**HR (95% CI)**	***P* value**	**HR (95% CI)**	***P* value**
**MetS status**						
No MetS	1(Reference)		1(Reference)		1(Reference)	
MetS	1.12 (1.09-1.16)	<0.001	1.10 (1.07-1.14)	<0.001	1.07 (1.03-1.10)	<0.001
**No. of MetS traits**						
0	1(Reference)		1(Reference)		1(Reference)	
1	0.98 (0.92, 1.05)	0.626	0.97 (0.91-1.04)	0.403	0.98 (0.92-1.04)	0.506
2	1.06 (1.00, 1.13)	0.049	1.05 (0.98, 1.11)	0.163	1.04 (0.98, 1.11)	0.188
3	1.12 (1.05, 1.19)	<0.001	1.09 (1.02, 1.16)	0.009	1.07 (1.00, 1.15)	0.04
4	1.18 (1.10, 1.26)	<0.001	1.14 (1.06, 1.22)	<0.001	1.11 (1.03, 1.20)	0.009
5	1.29 (1.18, 1.42)	<0.001	1.24 (1.13, 1.36)	<0.001	1.19 (1.07, 1.31)	<0.001
**Individual MetS components**						
Elevated WC	1.12 (1.08, 1.15)	<0.001	1.11 (1.07, 1.14)	<0.001	1.08 (1.03, 1.12)	0.001
Elevated TG	1.02 (0.99, 1.05)	0.132	1.02 (0.99, 1.05)	0.157	1.00 (0.97, 1.03)	0.883
Elevated BP	0.99 (0.96, 1.03)	0.779	0.98 (0.94, 1.02)	0.289	0.97 (0.94, 1.01)	0.177
Elevated HbA1c	1.15 (1.10, 1.20)	<0.001	1.12 (1.07, 1.17)	<0.001	1.07 (1.02, 1.12)	0.003
Reduced HDL	1.18 (1.14, 1.21)	<0.001	1.16 (1.12, 1.19)	<0.001	1.12 (1.09, 1.16)	<0.001

Model 1 adjusted for no covariates. Model 2 adjusted for age, ethnicity, smoking status, socioeconomic status, and Townsend's deprivation index. Model 3 adjusted for model 2 plus BMI, smoking status, drinking status, physical activity, and healthy diet score

HR – hazard ratio, CI – confidence interval.
